# Facets and mechanisms of adaptive pain behavior: predictive regulation and action

**DOI:** 10.3389/fnhum.2013.00755

**Published:** 2013-11-28

**Authors:** India Morrison, Irene Perini, James Dunham

**Affiliations:** ^1^Department of Clinical Neurophysiology, Sahlgrenska University HospitalGothenburg, Sweden; ^2^Institute of Neuroscience and Physiology, University of GothenburgGothenburg, Sweden; ^3^Department of Cognitive Neuroscience and Philosophy, University of SkövdeSkövde, Sweden; ^4^Division of Anaesthesia, University of Cambridge, Addenbrookes University Hospital, Cambridge University Hospitals NHS Foundation TrustCambridge, UK

**Keywords:** nociception, pain, action, allostasis, predictive regulation

## Abstract

Neural mechanisms underlying nociception and pain perception are considered to serve the ultimate goal of limiting tissue damage. However, since pain usually occurs in complex environments and situations that call for elaborate control over behavior, simple avoidance is insufficient to explain a range of mammalian pain responses, especially in the presence of competing goals. In this integrative review we propose a Predictive Regulation and Action (PRA) model of acute pain processing. It emphasizes evidence that the nervous system is organized to anticipate potential pain and to adjust behavior before the risk of tissue damage becomes critical. Regulatory processes occur on many levels, and can be dynamically influenced by local interactions or by modulation from other brain areas in the network. The PRA model centers on neural substrates supporting the predictive nature of pain processing, as well as on finely-calibrated yet versatile regulatory processes that ultimately affect behavior. We outline several operational categories of pain behavior, from spinally-mediated reflexes to adaptive voluntary action, situated at various neural levels. An implication is that neural processes that track potential tissue damage in terms of behavioral consequences are an integral part of pain perception.

“…we need to re-examine whether pain signals the presence of a stimulus, or whether it signals a stage reached in a series of possible actions.”

*Patrick Wall, 1999 (p 155)*

## Introduction

Even at the earliest stages of cortical processing, it is difficult to view pain processing as a strictly sensory description of a stimulus. Rather, the processing of nociceptive information in the spinal cord, brainstem, and subcortical pathways convey to the cortex a history of multiple sensorimotor transformations, ranging from reflex action to modulatory feedback. By the time a pain-relevant signal reaches the cortex, if not before, the terms of “nociceptive processing” become inadequate to describe pain representation, just as the terms of “auditory processing” become inadequate to describe music. Evidence from many strands of current pain research suggest that from the very outset, pain processing deals with complex, nested representations of relationships between stimulus and action.

In this review we consider pain not primarily as a sensation, but as an action problem. In this perspective, a nociceptive signal travelling from the periphery via the spinal cord presents the brain with the question “what is to be done?” We propose a Predictive Regulation and Action (PRA) model of pain, which incorporates evidence that the organization of pain system is inherently action-centered, at levels from the spinal cord to the cortex. In this model, an emphasis on pain as a sensory signal is relieved, in favor of an emphasis on dynamic sensorimotor transformations among multiple interacting systems, each jostling to offer solutions to the problem of “what is to be done” when potential injury looms.

## Predictive Regulation and Action (PRA) model of pain

As the name suggests, the PRA model of pain seeks to capture several key aspects of pain processing: prediction, regulation, and action. The prediction component brings out the idea that neural subsystems operate not just on the basis of actual signals from communicating subsystems, but on their dynamic predictions of such signals in hierarchically-organized networks (Clark, 2013). Such cascades of multiple predictions introduce the need for regulatory processes, both local and supervening, which handle error signals, assign signal weights, as well as influencing gain-modulation in other parts of the system, in pursuit of stable and energy-efficient processing. Finally, the PRA model considers pain processing as ultimately geared towards behavior. In particular, much of cortical pain processing reflects the modulation of voluntary actions in response to pain, within systems that take into account multidimensional information such as context, memory, rule-based contingencies, and even efference from past spinal reflex actions.

The PRA model synthesizes theoretical and computational ideas from the domains of action and regulatory control. Core elements of these models will be covered in more detail in later sections, and we direct the interested reader to the cited papers for formalizations of these ideas (especially Sterling, 1988; Koechlin and Summerfield, 2007; Clark, 2013; Shenhav et al., 2013). For our present purposes, we first highlight several common themes that emerge from them.

The first is *prediction*, which has become a central concept in many current models of action, cognition, and emotion. Predictive coding also has important implications for the way *error* signals are handled in the system. Regulatory processing that generates and handles such prediction and error information in turn invokes the idea of *energy-efficiency*, in which the dynamics of a neural system tend to stabilize around operations that utilize available metabolic energy with as little waste as possible. Towards that end, adaptive *tradeoffs* may occur within the system. These tradeoffs are at least partly constrained by processes predicting likely *costs and benefits*. The bottom line of predictive regulation is *behavior*. The synthesis of these ideas in the PRA model is intended to capture numerous features of the nervous system’s organization that allow the anticipation of potential pain, adaptive adjustment of behavior, and the management of energetic costs—all before the risk of tissue damage becomes critical.

## Withdrawal reflex action and predictive adjustment of behavior

Withdrawal is probably the action type most frequently associated with the acute pain of injury. Such rapid, involuntary limb withdrawal actions are supported by spinal reflexes, which are in turn triggered by nociceptor activation. In the laboratory, standard tests involve measuring the latency of an animal’s limb withdrawal from a hot (Hargreaves et al., 1988) or cold (Jasmin et al., 1998; Allchorne et al., 2005) plate at or above-threshold intensity (usually in rats or mice). The formalin test, which involves chemically injuring tissue by formalin injection, is another standard protocol for probing withdrawal and protective behaviors (Dubuisson and Dennis, 1977).

Heat, cold, and mechanical stimuli elicit withdrawal or flexion reflexes in both awake (Chaplan et al., 1994; McMullan et al., 2004; Dunham et al., 2010) and anesthetized animals (Bessou et al., 1971; Yeomans et al., 1996). C heat fibers (including polymodal C fibers) and Aδ fibers underlie the initial encoding of a noxious heat stimulus (Dunham et al., 2010), and Aδ fibers also signal noxious skin deformations from mechanical stimuli (Bessou et al., 1971; Lewin and Moshourab, 2004). In contrast to heat- or mechanically-mediated withdrawal behavior, cold-mediated withdrawal may be more dependent upon differing activity in multiple populations of afferents (Campero et al., 1996; Simone and Kajander, 1996, 1997). To our knowledge, electrophysiological data more definitively linking particular nociceptor populations to withdrawal to cold temperatures is lacking (for cold withdrawal behavior see Dunham and Donaldson, 2007).

Yet the relationship between tissue damage and nociceptor activation is not straightforward. Nociceptor activation does not necessarily signal tissue damage—rather, it signals a *risk* of tissue damage. This is partly owing to a gap between the point at which nociceptor classes in the skin reach their firing threshold, which is relatively invariant (for example, around 38°–42°C for heat nociceptors), and the point at which actual tissue damage occurs (for example, denaturation of tissue proteins starts at about 45°C). This liberal setting of nociceptor thresholds effectively exaggerates an input signal in order to provoke pre-emptive behavioral responses (Raja et al., 1999). The system is biased to react as if injury has actually occurred, because non-damaging degrees of stimulation in this range reliably and probabilistically predict actual tissue damage. So starting at the first stage of sensory response, at the afferent nerve level, nociception already reflects a predictive, probabilistic risk assessment. Thus, reflexes elicited by nociceptor activation are frequently protective. In this sense they are comparable to representations of metabolic need, in which hunger feelings and motivation to eat precede critical metabolic deficiency in the body’s tissues.

Such protective withdrawal reflex actions are mainly supported by neurons in the spinal cord that receive signals from nociceptors. “Nociceptive withdrawal reflex” (NWR) neurons mediating muscle activations have been identified in the dorsal horn of the spinal cord (Levinsson et al., 2002). One might expect that these spinal NWR circuits are organized with respect to a somatosensory map of the incoming sensory afferent sources, but evidence indicates that they are not organized in this sensory-afferent-based manner. Instead, they are mapped with respect to the target muscle (Schouenborg and Weng, 1994; Sonnenborg et al., 2000; Levinsson et al., 2002; Schouenborg, 2003). This musculatopic mapping implies that NWR circuits are tuned to optimize sensorimotor transformations of incoming nociceptive information in the *efferent* direction, in terms of their influence on the specific muscles they innervate. Encoding of nociceptive signals is thus action-based from a very early stage.

Non-nociceptive tactile information may also be utilized in circuits that control pain-withdrawal behaviors. Most NWRs are wide-dynamic range neurons (WDRs) in the dorsal horn of the spinal cord. WDRs receive input from a variety of tactile afferents, both nociceptive and non-nociceptive. Such neurons in NWR circuits may weight tactile afferent input from the receptive field alongside nociceptive input, suggesting that non-nociceptive tactile information is taken into account in reflexive withdrawal action (Petersson et al., 2003). It is currently unclear whether non-nociceptive tactile information influences the production of a given instance of reflex withdrawal, but work with rats and cats indicates that input from non-nociceptive tactile afferents may be crucial in setting the gain on NWR circuits in the spinal cord (Holmberg and Schouenborg, 1996). Specifically, spontaneous muscle twitches during sleep (when the sensory background is otherwise relatively quiet) result in tactile signals from the skin to the NWR spinal reflex circuit (Holmberg and Schouenborg, 1996; Petersson et al., 2003; Waldenstrom et al., 2003). The NWR may apply these signals to tune the efficacy of muscle action with respect to skin sensation. In other words, feedback from low-threshold tactile mechanoreceptors can help NWR circuits encode how effectively a particular muscle contraction can “unload” an offensive stimulus from the tactile receptive field. This process has been named “somatosensory imprinting” (Holmberg and Schouenborg, 1996; Waldenstrom et al., 2003).

Somatosensory imprinting can be considered a gain-modulating mechanism influencing the efficiency of efferent output to the muscle, in essence putting innocuous tactile information to use in order to fine-tune reflex actions. Non-nociceptive tactile afferent input may be sufficient to activate NWR units to produce behavioral adjustments in the *absence* of nociceptor input, as suggested by inhibition of the RIII withdrawal reflex by innocuous electrical stimulation of skin over the specific nerve pathway (Danziger et al., 1998). But it is possible that pain-processing systems are open to coding non-nociceptive information in pain-related terms, if it predicts a probable ramp-up to nociceptor activation. In rats, gradually decreasing cold stimulation has been observed to provoke a flick response on the stimulated foot—but at temperatures insufficiently cool to excite large numbers of cold-sensitive nociceptors (Dunham and Donaldson, 2007). Following injury, innocuous stimulation around the injury site can also produce unpleasant or painful sensations (Chaplan et al., 1994), which may in part arise from sensitization of afferent neural populations in the spinal cord (Liljencrantz et al., 2013).

We coin the term “protonoxial adjustment” for non-injury-related behavioral adjustments that occur in the presence of innocuous stimuli which are not sufficiently strong in themselves to surpass nociceptor thresholds. For example, holding a cool drink in one hand might cause enough discomfort for you to change hands after a while, despite not being cold enough to evoke a nociceptor response. Such protonoxial adjustment behaviors may partly rely on mechanisms in the central nervous system which predict somaesthetic perturbations by innocuous stimuli on the basis of previous experience (such as the eventual local numbness from holding a cold drink for too long). However, this remains to be experimentally addressed.

## Complex nocifensive behavior

Spinally-mediated and autonomic reflexes (such as withdrawal and startle, respectively) go far in accounting for the first wave of bodily defense and action readiness in the face of potential pain. However, mammalian cortex supports complex mechanisms for further flexibility and refinement of action, integrating reflex responses with higher-level spatial, temporal, and sensory information. Once again, these processes often occur in a predictive manner.

Nocifensive actions, such as swatting at a particular location with an arm using a particular force, require visuotactile and spatiotemporal integration of pain-related information as well as its sensorimotor transformation. Neural populations in primate posterior parietal cortex perform sensorimotor transformations of threat-relevant visual stimuli (Rizzolatti et al., 1997; Buneo et al., 2002; Calton et al., 2002; Fogassi and Luppino, 2005). This can occur because many neurons in these populations are “bimodal”, responding to both tactile and visual stimuli in a common receptive field, for example on the cheek skin and the area of space near the cheek. In ventral intraparietal sulcus (VIP), part of the frontoparietal action circuit, microstimulation produces appropriate eye, lip, and arm movements similar to those elicited by an aversive airpuff into the eyes (Cooke and Graziano, 2003). Human parietal cortex may similarly encode aversive visual events within peripersonal hand space (Lloyd et al., 2006), indicating a role for the VIP in the orchestration of aversive movements that require integration of visuotactile information into an egocentric coordinate frame (Graziano and Cooke, 2006). Importantly, coding of a stimulus in the space *near* the face in the same terms as one actually *touching* the face can be seen as a predictive mechanism which treats spatial information on a par with tactile information. These parietal populations have anatomical connections to posterior cingulate cortex (PCC), which plays a central role in orienting the eyes and body towards threatening stimuli (Vogt et al., 2006).

Among populations in nearby parietal area 7b (macaque homologue of human area PF) are also pain-related sensory neurons that also show visual response properties, which fire both when a part of the skin on the face is stimulated with noxious heat, and when the monkey views a threatening stimulus coming towards or hovering near that part of the skin (Dong et al., 1994). In humans, meta-analysis of fMRI studies has shown that PF and surrounding inferior parietal cortex are commonly activated by painful stimuli and action execution tasks (Morrison et al., 2013), including facial expression (Budell et al., 2010), consistent with a close yoking of pain information with action planning and execution. Predicting the probable sensory consequences of an action may thus be part of the package of action planning, for example, in reaching and grasp formation (Morrison et al., 2013).

The human hand-blink reflex (HBR) illustrates the complex interaction between nocifensive responses and the spatial representation of the envelope of peripersonal space surrounding the body. If the arm’s median nerve is stimulated as the hand is brought rapidly towards the face, this elicits an eyeblink reflex (Sambo et al., 2012a). The coordination of hand stimulation with a trigeminally (i.e., facial nerve) mediated eyeblink response reflects the integration of proprioceptive information (here, from the hand) with the coding of peripersonal space (here, around the face). An electromyographic (EMG) study has shown that the HBR is enhanced most when the hand is within peripersonal space and nearest the face (Sambo et al., 2012b). It is also specific to the relevant hand and dependent on cognitive expectations (Sambo et al., 2012b). This brainstem-level coding of “defensive” peripersonal space is dynamic and facilitates appropriate nocifensive action before an actual injury occurs (Sambo et al., 2012a). Further, transcranial magnetic stimulation (TMS) of motor cortex has revealed evidence of complex interactions among arm and hand muscles during pain, with reduced muscle-evoked potentials (MEPs) in distal (hand) muscles alongside a slight facilitation of proximal (upper arm) muscles, which likely reflect enhanced arm retraction simultaneously with prehension interruption (Leis et al., 2000; Le Pera et al., 2001).

## Predictive coding and weighting of risk estimates

Predictive coding in the nervous system can take many forms. Since nociceptor activation has reliably predicted tissue damage during phylogenetic history, it can be considered a signal of tissue damage risk. In the nocifensive action circuits just discussed, temporal and spatial information about events around and within the body envelope predict potential threat, and reflect complex sensorimotor integration of such predictions. Multiple neural connections bear such sensory-based signals forward among the intricately nested hierarchy of systems involved in pain processing.

Yet crucially, pain processing is not a feedforward affair. Especially in the cortex, “backward” connections can operate on incoming signals to modulate their strength or salience. For example, descending modulation can attenuate the incoming nociceptive signal from the spinal cord (Fields et al., 1977; Calejesan et al., 2000), effectively skewing the input range away from higher stimulus extremes. Pain behavior thresholds of laboratory animals can be influenced by contextual factors, such as the identity of the experimenter handling them (Chesler et al., 2002). In humans, voluntary attentional focus (Ploghaus et al., 1999; Kulkarni et al., 2005), expectation (Wiech et al., 2008), and contextual factors (Rudy et al., 2004; Jepma and Wager, 2013), and social factors (Krahé et al., 2013) can bias cortical pain processing. Spinal-level effects of descending modulation of pain by attention (Sprenger et al., 2012) and by negative emotion (Rhudy et al., 2013) have recently been demonstrated in humans.

In the PRA model, such back-modulating regulatory processes pivot on local predictions about the incoming signal. If an incoming signal to a given neural population deviates from the predicted input signal, this generates a further, information-rich signal reflecting the residual error of the prediction. In turn, this gives rise to processes that seek to account for sources of the error within the system or network. This type of “dynamic predictive coding” model (Clark, 2013) has been fruitfully applied to perception-action systems (Grush, 2004; Friston, 2005) as well as interoceptive systems (Paulus and Stein, 2010; Seth et al., 2012).

One major implication is that the sensory-based stimulus information feeding such processes consists mostly of the forward propagation of informative *error* (rather than “sensory”) signals, while constantly-adjusted predictions propagate backwards and influence the forward flow of information. Rather than simply transducing the nociceptive signal, then, cortical pain networks mainly conduct their trading in the less expensive currency of error signals. In applying these ideas to pain, the PRA model implies that subjective pain experience involves the perception of this dance of prediction and error, rather than being a “direct perception” of nociceptive signaling.

The relative weighting of signals propagating through the network provides an estimate of risk, in that strongly-weighted nociceptive-based signals convey a higher likelihood of cost in terms of tissue damage. A high risk weighting also implies a high benefit of behavioral response. However, estimates may differ among different nodes of the system as to just how large a risk a given stimulus poses. For example, nociceptive signals from the spinal cord synapse in brainstem and thalamic nuclei before reaching the cortex, with information coded at each synapse along the way. Yet this forward chain of synapses probably over-estimates risk in order to guard against the perils of under-reaction. Recall the wide margin for nociceptor activation mentioned earlier: the signal is inherently exaggerated, with the needle swinging from “some likelihood of tissue damage” to “an actual injury has occurred” (even when none has). From an injury-avoidance perspective, this operational collapse of “potential” with “actual” injury is smart. From an energetic resource perspective, however, it is a recipe for unwarranted waste, since it sets up a costly false positive bias, perhaps all the way up to the thalamic level.

Allostatic models (Sterling, 1988; Schulkin, 2011) emphasize this kind of tension between ranges of prediction and energy-efficiency. For example, Sterling’s general allostatic model (Sterling, 1988) posits that stable dynamics reflect energy efficiency among multiple interacting systems, not necessarily defense against deviations from a given set point (in contrast to a more literal “homeostatic” model, see e.g., Schulkin, 2011). Like dynamic predictive coding models, allostatic models highlight the role of prior experience and prediction in the system’s maintenance of a stable dynamic. In these senses, an allostatic view is well-equipped to describe important features of the complex, multivariate mechanisms of regulation among the multiple interacting systems involved in pain processing (such as inflammation and stress; see also Maleki et al. (2012) for an application to migraine pain). Thus, risk weighting may provide a spur to action to other parts of the system, but other parts of the system can also play a role in deciding how seriously to take the risk estimate when the error signal is large. This dynamic should converge on energy-efficient interactions within the system, ultimately influencing the deployment of behavior in response to the nociceptive signal.

At the cortical level, incoming overestimations of risk would result in high error signals, leading to re-estimates of risk in the face of experience and other supervening contextual information, as well as re-weighting of signals to reflect error correction. The backward flow of regulatory influence can be understood in terms of gain modulation. Increasing the gain on an incoming risk estimate signal may effectively heighten the signal’s salience with respect to ongoing action priorities and behavior. Likewise, lowering the gain through regulatory re-weighting can adjust its salience with respect to high-level factors, particularly where action is potentially costly. This relationship between prediction and regulation holds not only for ongoing stimulus processing but also affects predictions for future situations.

**Figure 1:**
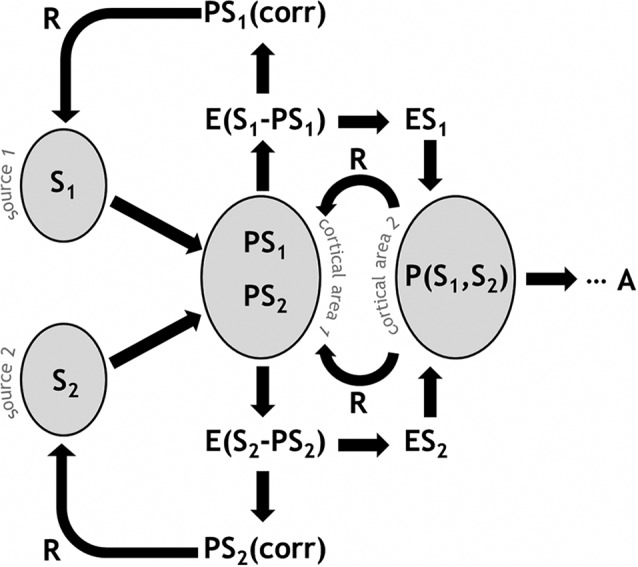
**Schematic illustration of predictive coding and error-based regulation in the PRA model of pain processing.** A given cortical area receives signals (S) from multiple input sources. Predictions (PS) in the neural population represent the expected input values. An error signal (E) arises from the disparity between S and PS. A PScorr feeds back to previous stages. Higher-order predictions are likely to take multiple signal sources into account, e.g., *P*(S_1_, S_2_). Ultimately, this process results in a behavioral response or action (A).

Figure 1 presents a schematic illustration of predictive coding and error-based regulation in a simplified system. A cortical area receives signals (S) from multiple input sources, for example from different thalamic nuclei. Predictions (PS) in the form of particular neural configurations await the signal, sketching out the expected input values. The differential between S and PS generates an error signal (E). Depending on the information to which the network has access, the signal variance is partially explained and therefore reduced, with a corrected prediction (PScorr) feeding back to previous stages or even to the signal sources themselves. As a whole, this process is regulatory and effectively gain-modulating, for example re-weighting the risk estimate reflected by S. Importantly, cortical populations integrate information, so higher-order predictions are likely to take multiple signal sources into account, e.g., *P*(S_1_, S_2_). This also means that backward-propagating regulatory information is comparatively refined with respect to multiple signal sources. Ultimately, this process results in a behavioral response or action (A).

**Figure 2:**
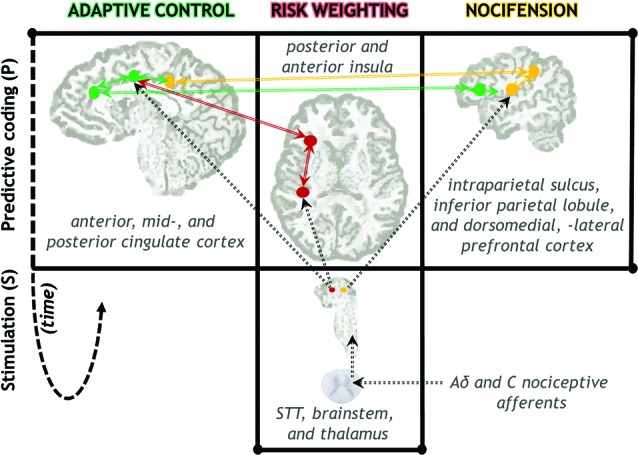
**Neuroanatomical diagram of key neural circuits in the PRA model of pain processing.** Signals from nociceptive afferents arrive in cortex via STT, brainstem, and thalamic nuclei. The insula plays a central role in comparing these signals to predictions, as well as regulatory feedback processes that gain-modulate incoming signal weights (red; see also Figure 1). Spatiotemporal and proprioceptive information is integrated with pain information in parietal-centered circuits supporting nocifensive behavior (yellow). Voluntary actions in the face of actual or potential injury are supported by prefrontal regions, in particular anterior and mid-cingulate cortex (green). Action selection is likely to be influenced by risk-relevant information from the insula. Solid arrows indicate anatomical connectivity among highlighted regions. Dotted lines indicate selected major anatomical projections from the STT via thalamus. The time arrow (dotted fish-hook) indicates both the predictive (pre-stimulus) nature of these representations as well as the reiterative nature of the regulatory gain-setting processes. (Note: this schematic does not show all nociceptive-relevant regions and projections.)

In the PRA model, the posterior insula is a main hub (Pessoa, 2008) not only for receiving nociceptive-based signals from thalamus (Figure 2), but integrating this information into subjective (Craig, 2003a,b; Paulus, 2007) and autonomic efferent terms (Damasio, 2000; Critchley et al., 2004; Gianaros et al., 2012). Most nociceptive afferents from the skin follow the spinothalamic tract (STT) to the cortex. Evidence from nonhuman primates suggests that posterior insula is one of the major projection sites of the STT, via thalamic nuclei containing nociceptive neurons (Craig and Zhang, 2006; Dum et al., 2009). Intriguingly, this is the only pain-related cortical region that produces subjective sensations of pain when directly electrically stimulated (Bancaud et al., 1976).

The anterior insula is also likely to be a closely-related partner in these cortical re-weighting processes, handling error signals based on thalamocortical inputs (for a similar idea see Refs. Damasio, 2000 and Craig, 2009). Processes of integrating and re-evaluating risk estimates may follow a caudo-rostral “gradient” in the insula, reaching a high degree of integration at the anterior insula (Craig, 2009). This gradient shows corresponding gradual caudo-rostral shifts in terms of connectivity with other cortical networks (Cerliani et al., 2012). Recent human neuroimaging evidence suggests that anterior insula activity predicts whether a subject will classify a stimulus as painful, biasing “perceptual decisions” about pain even before the stimulus occurs (Wiech et al., 2010). Importantly, this suggests a predictive relationship among nociceptive signals and insula processing, rather than a feedforward sequence of information handling. Patients with insular lesions evaluate pain as more intense on their affected side, suggesting that weighting is altered when insula is damaged, and show a greater recruitment of somatosensory cortices contralateral to the lesion, suggesting less efficient modulatory dynamics in these processes (Starr et al., 2009). Gain-modulation mechanisms in insula may contribute to pain’s subjectively “hot” stamp by influencing signal salience (Mouraux et al., 2011).

Anterior insula has at least two subdivisions, a ventral, agranular region associated with affective processing and interconnected with many classical limbic structures such as the amygdala; and a dorsal, dysgranular region showing anatomical and functional connections with parietal and cingulate networks (Kurth et al., 2010; Wiech et al., 2010; Touroutoglou et al., 2012). Interestingly, there is also a degree of overlap between these two areas in terms of their intrinsic (resting state) connectivity (Kurth et al., 2010), suggesting scope for close functional communication between these insular subregions and their associated networks. In the rat (unlike in humans), rostral agranular insular cortex receives direct input from nociceptive neurons in medial thalamic nuclei. Specific gain-setting mechanisms of the pain signal may operate here, with gamma-aminobutyric acid (GABA) dynamically modulating neural thresholds to dampen or heighten pain behavior (Jasmin et al., 2003). Although the rat’s gross neuroanatomy differs from the human’s in agranular anterior insula, the neurotransmitter mechanisms mediating gain-setting of pain signals may be similar.

The PRA model accommodates an important aspect of allostatic regulation within predictive coding networks: uncertainty. In normal acute pain, high S and high P produce low error signals and low modulatory regulation, passing a relatively unfiltered high risk estimate on through the system with high certainty. When there is no tissue damage risk at all, error and regulation are low, passing on a relatively unfiltered low risk estimate with high certainty. However, mismatches between S and P can produce high error and high uncertainty, if the system is unable to “explain away” much of the error residual.

High uncertainty alongside appropriate regulation might bootstrap learning in some circumstances, for example during the acquisition of conditioned pain responses (e.g., Rudy et al., 2004) or the reversal of such conditioning (e.g., Schiller and Delgado, 2010). Some pain syndromes and pathological pain conditions may involve high Ps in the face of relatively low weightings of S, thus overestimating risk (e.g., nocebo hyperalgesia, Colloca and Benedetti, 2007). Hypoalgesia or attenuated pain behavior may involve low Ps in the face of high weightings of S. All of these processes also have a vital temporal dimension, with failure to regulate in the right way at the right time leading to potential dysfuntion. In this perspective, anticipation of pain and pain anxiety are outcomes of high Ps, which may or may not be appropriately corrected either by bottom-up Ss (such as nociceptor activation or afferent sensitization at the spinal level) or by top-down regulatory P (corr) processes (as in descending modulation or episodic learning). Such processes could be involved in complex pain-emotion relationships like fear conditioning and the extinction and regulation of fear responses in the face of pain (Colloca and Benedetti, 2007; Schiller and Delgado, 2010; Rhudy et al., 2013).

## Adaptive control processes in the cortex

Strongly-weighted pain signals in the cortex can very effectively disrupt existing goals and override their associated behaviors. Yet no matter how strongly a given risk signal is estimated in the system, simple avoidance action is insufficient to explain all pain behavior. In many circumstances, pain’s ultimate function of limiting tissue damage (e.g., Merskey and Bogduk, 1994) becomes complicated by the need to balance incoming nociceptive-based information with current goals and states. Sometimes the conflict is easy to resolve. For example, extreme heat on unprotected skin represents such an immediate tissue damage threat that it elicits spinal reflex action when you grasp a hot pan. But when tissue damage is more a vivid prospect than a reality, the relative weighting of sensations and goals is more difficult to resolve and requires more finely-calibrated control of behavioral outcomes.

Cortical pain representation may get a boost from the action-based pre-packaging of the incoming signal discussed earlier, in which nociceptive information is coded in sensorimotor terms even at the spinal level. But why should there be a need for further control? Frequently, there isn’t. Often the sensorimotor information is sufficient for producing an appropriate action, and many risk-weighted events lead to straightforward avoidance or protective behavior. In other words, predictive coding explains and gain-modulates input signals so well that little residual error remains to propagate through the system. However, cortical representations of nociceptive signals arrive on a scene that is already bustling with various goals and motivational states—which may or may not have bearing on the question of how to act on the pain (see Chaplan et al., 1994 for an exploration of this idea with respect to opioid modulation; see also Fields, 2007). Generally speaking, the role of the cortex is to handle additional levels of conditional information that may be relevant to action selection in the face of pain. The high degree of flexibility which these processes confer arises from what Shackman and colleagues (Shackman et al., 2011) have termed “adaptive control” mechanisms in the cortex.

Such systems can be formally characterized as executive systems. The conditional information that cortical executive systems handle can be viewed as a hierarchically organized “cascade” of tightly interlinked levels (Fuster, 1991). In Koechlin and Summerfield’s influential model of premotor executive control (Koechlin and Summerfield, 2007), the action information that comes bundled with the stimulus information occupies a basic level in the hierarchy, in which there is negligible residual between predicted sensory and motor signals. A further level of control subsumes both immediate context and episodic memory of past events. A still further level incorporates any relevant rule-based or otherwise contingent (e.g., “if-then”) information that entails entertaining many possible action outcomes simultaneously. Applying this to the PRA framework, the greater the amount of additional information needed to select an appropriate action in the face of pain—or the greater the error—the greater the demand for higher-order levels of executive control.

**Figure 3:**
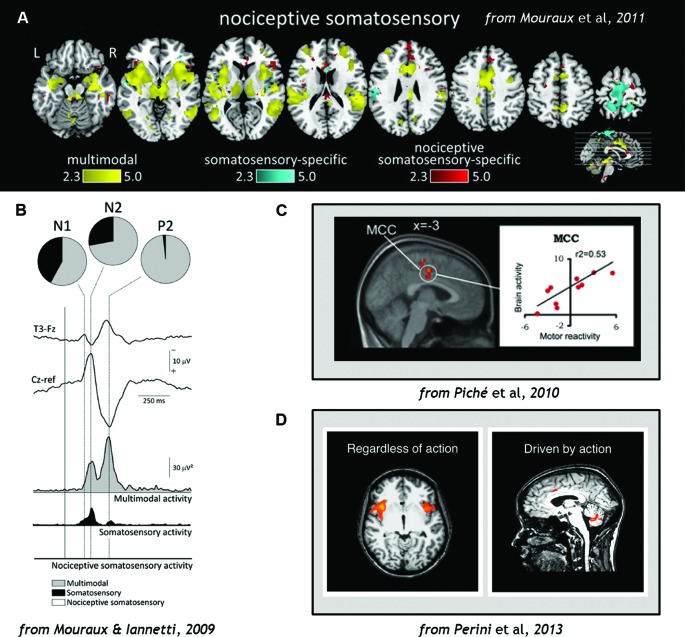
**(A)** Analysis of fMRI data comparing blood-oxygen-level-dependent (BOLD) responses to stimulation across sensory domains (vision, audition, somatosensation, and pain), indicating that multimodal activity accounts for most of the cortical network activation during pain (Wiech et al., 2010). **(B)** EEG results demonstrating that cortical responses to Aδ and C nociceptor activation by laser-evoked potential (LEP) stimulation reflects predominantly multimodal and to some extent somatosensory–specific activity, but limited nociceptive-specific activity (Fuster, 1991). **(C)** fMRI evidence demonstrating correlations in regions of midcingulate cortex (MCC) with individual motor reactivity in the spinal RIII reflex (top) and variance in autonomic arousal (bottom) during electrical pain stimulation (Bancaud et al., 1976). **(D)** fMRI evidence demonstrates that midcingulate but not anterior insula activations during pain are contingent on motor processing (Duerden and Albanese, 2013). Figures reproduced with permission.

A recent activation-likelihood-estimate (ALE) meta-analysis of fMRI studies showed that the anterior cingulate cortex (ACC) is the region most likely to be activated by acute pain (Duerden and Albanese, 2013). Although the cingulate cortex is often regarded as a key area in a “pain neuromatrix”, a specific role in pain is unlikely, since it is also implicated in a range of non-pain-related functions (Mouraux and Iannetti, 2009). At the cortical level, even somatosensory contribution to pain processing may be small, and nociceptive-specific contribution even smaller, compared to multimodal processing in networks throughout the brain (Mouraux and Iannetti, 2009; Mouraux et al., 2011; Figure 3).

We consider the involvement of medial areas such as midcingulate cortex (MCC) far more likely to involve the adaptive control of action during pain (Shackman et al., 2011; Perini et al., 2013). Primate medial wall cortical areas including the ACC and MCC contain premotor fields (cingulate motor zones) which have both output to and input from cervical segments of the spinal cord where motoneurons are located (Picard and Strick, 1996; Koski and Paus, 2000; Dum et al., 2009), suggesting that these areas play a role in the generation and control of movements (Matelli et al., 1986; Picard and Strick, 1996; Koski and Paus, 2000; Dum et al., 2009; Perini et al., 2013). Like the posterior insula, they receive projections from the STT (Dum and Strick, 1996). But unlike posterior insula, intracranial microstimulation of human ACC does *not* result in pain sensations, but in reported feelings of urgency (Bancaud et al., 1976; Hsieh et al., 1994). Indeed, as the duration of a painful thermal stimulus increases, so do subjects’ ratings of their urge to move away from the stimulus (Perini et al., 2013).

Regions of ACC and MCC have also been implicated in individual variance in motor reactivity, with nearby areas tracking autonomic variance (Piché et al., 2010; Figure 3). ACC responses to noxious thermal stimuli in the macaque monkey have shown increased activity during voluntary escape responses (in which monkeys could push a button to end the painful stimulation without performing the rewarded detection task; Iwata et al., 2005). However, these neurons showed decreased activity to the same stimulation during illumination and temperature change-detection tasks which required suppression of any immediate motor responses to the pain (Iwata et al., 2005). This indicates that the same region of the brain can mediate facilitory or inhibitory control over motor responses during pain. Recent human neuroimaging evidence indicates that voluntary motor-related processing can account for MCC and ACC activation during pain, particularly in the caudal cingulate motor zone (CCZ; Perini et al., 2013; Figure 3).

Kochelin and Summerfield’s model of executive control can be applied to the medial prefrontal networks in which ACC and MCC are central hubs (Kouneiher et al., 2009). The ACC in particular has been extensively implicated in control-related processing across a range of contexts (Shenhav et al., 2013). The cingulate cortex is therefore a major site of executive control processes underlying adaptive control of pain behavior (Shackman et al., 2011; Duerden and Albanese, 2013). These often display a caudal-rostral gradient (as do premotor executive control processes elsewhere in cortex), indicating that ACC and MCC subregions work together to integrate stimulus content and current task demands to produce appropriate and timely responses (Vogt, 2005; Kouneiher et al., 2009). Its role in such functions is partly owing to processes that link predicted value comparisons with action choices (Rushworth et al., 2012; Demanet et al., 2013). In this sense it is likely to be heavily involved in dynamic predictive representation of pain-relevant information.

Most caudally, the dorsal posterior cingulate cortex (dPCC) receives inputs from dorsal-stream parietal areas implicated in nocifensive behavior (Graziano and Cooke, 2006) and is also involved in orienting to and organizing motor responses to pain (Vogt et al., 2006). The motor fields of MCC probably contribute heavily to mobilizing context-appropriate skeletomotor responses to pain, with hemodynamic responses in the CCZ correlating with reaction times to pain (Perini et al., 2013). This region is also related to the regulation of facial expression displays during pain (Kunz et al., 2011). Importantly, neuroimaging analysis incorporating reflex variance indicates that it also receives its own “copy” of spinal reflex efference. The human RIII reflex is involved in limb withdrawal following nociceptive input to the spinal cord, and is measured by EMG activity from the muscle. Within-subject variability in human RIII reflex thresholds during painful electrical stimulation were associated with BOLD modulation of the MCC and ACC (Piché et al., 2010; see also Figure 3). These regions also get their own “copy” of the nociceptive signal from the STT from the same thalamic populations that project to posterior insula (Dum et al., 2009; Figure 2). They are also associated with the affective dimension of pain (Rainville et al., 1997).

Most rostrally, the rostral cingulate motor zone (RCZ) may be enlisted when the situation involves more complex conditional information, such as increased task complexity or dimensionality (Kouneiher et al., 2009). Processing in ACC may encode current and alternative courses of action, privileging some options in a manner closely linked to motivated choice and exploration behavior (Bancaud et al., 1976). Rostral ACC regions are particularly densely interconnected with dorsomedial and dorsolateral prefrontal networks also implicated in executive processing and action selection. These areas may contribute to a ranking of choices in both current and prospective temporal windows, perhaps even interacting in a competitive manner (Rushworth et al., 2012; Demanet et al., 2013). In the PRA model perspective, such action-based predictions might even stand in for sensory information under certain circumstances. Indeed, human psychophysical evidence suggests that, with practice, motor-based coding can improve sensory acuity without any changes in the sensory input (Saig et al., 2012).

Both adaptive control and risk-estimate-reweighting are interacting regulatory processes constrained by factors impinging on energy efficiency (Sengupta et al., 2013). Often, this involves weighing costs and benefits. Predictive systems can “look ahead” and project potential costs and benefits of outputs (ultimately, behavior) with respect to the signals from a variety of domains, including pain. For example, a rat may continue to forage for food in subzero temperatures, because the expected metabolic benefit of eating probably outweighs the current risk of tissue damage from the cold (Cabanac and Johnson, 1983; Boorman et al., 2013). Evidence from human behavior suggests that the magnitude and probability of painful stimulation can guide human behavior in a relatively direct manner (Kurniawan et al., 2010). But these mechanisms can also show complex sensitivity to previous experience, as well as any “market forces” that assign a reward value to pain tolerance (Vlaev et al., 2009), or by other outcomes which offset threat aversiveness (Hu et al., 2013). Under certain circumstances, the nociceptive route to cortex might even bypass somatosensory cortices, as suggested by a novel analysis (Liang et al., 2013), raising the possibility that sometimes even detailed somatosensory processing of a nociceptive signal can carry a prohibitively high cost. Such evidence for cost-benefit analyses with respect to pain behavior is consistent with allostatic and adaptive control processes that allow simple avoidance behavior to be circumvented in favor of expected benefits, especially those involving goals from other domains or the higher-order prospective goals we humans specialize in.

It is important to emphasize that we do not consider adaptive control processes as divorced from pain perception or its subjective nature. On the contrary, the PRA model postulates that adaptive action control processes are partly *constitutive* of subjective acute pain experience (Perini et al., 2013). We speculate that whereas predictive, regulatory processes producing risk estimate signals (as in the insula, Figure 2) probably make a large contribution to acute pain perception, so do movement urges arising from the action control hierarchies that both utilize and gain-set those risk signals (as in the cingulate, Figure 2). The interacting cingulate subsystems recruited by pain, for example, are both goal-directed and “energized” by risk and error signals originating in insula, among other places (for a detailed neurocomputational view see Holroyd and Yeung, 2012; Rushworth et al., 2012).

## Conclusion

The PRA model of acute pain processing is an action-centered pain model that takes into account predictive coding, handling of error signals, local and supervening regulation, and dynamic interactions among the myriad hierarchical systems involved in processing acute nociceptive signals from the periphery. It also delineates operational categories of pain behavior. On the cortical level, the model focuses on the roles of the insula and the cingulate in gain-setting and action selection processes during pain. The insula may be involved in re-weighting the tissue-damage-risk estimates carried by thalamic nociceptive signals, possibly by dynamically setting the gain on nociceptive signal processing. Voluntary actions in the face of actual or potential injury are supported predominantly by MCC and ACC. The PRA model’s description of neuroanatomical systems is not exhaustive, but can serve as a backbone for the mapping of pain-related processes in the nervous system as a whole. It incorporates elements from dynamic predictive coding, allostatic, and executive control models which capture the predictive and dynamic nature of these processes.

## Conflict of interest statement

The authors declare that the research was conducted in the absence of any commercial or financial relationships that could be construed as a potential conflict of interest.
